# Clinical relevance of PLK1 in epithelial ovarian cancer

**DOI:** 10.3389/fphar.2025.1702273

**Published:** 2025-12-12

**Authors:** Xiaochang Shen, Jiandong Wang, Shuning Chen, Haomeng Zhang, Chunxiao Zhou, Victoria L. Bae-Jump

**Affiliations:** 1 Department of Gynecological Oncology, Beijing Obstetrics and Gynecology Hospital, Capital Medical University, Beijing Maternal and Child Health Care Hospital, Beijing, China; 2 Department of Obstetrics and Gynecology, Beijing Friendship Hospital, Capital Medical University, Beijing, China; 3 Division of Gynecologic Oncology, Department of Obstetrics and Gynecology, University of North Carolina at Chapel Hill, Chapel Hill, NC, United States; 4 Lineberger Comprehensive Cancer Center, University of North Carolina at Chapel Hill, Chapel Hill, NC, United States

**Keywords:** plk1, epithelial ovarian cancer, antitumor, pre-clinical model, clinical trial

## Abstract

Ovarian cancer, particularly epithelial ovarian cancer (EOC), is the most aggressive and lethal gynecologic malignancy, necessitating the development of innovative and potent therapies to improve its prognosis. Human polo-like kinase 1 (PLK1), a key regulator of cell division, is crucial for driving mitosis and cytokinesis and maintaining genomic stability through phosphorylation of a few different substrates. PLK1 is found to be overexpressed in EOC tissues compared with normal ovarian tissues and is strongly associated with the prognosis of patients with stage I and II EOC. Targeting PLK1 markedly inhibits cell proliferation, causes cell cycle G2 phase arrest and DNA damage, induces apoptosis, and reduces tumor growth in preclinical models of EOC. In addition, the combination of PLK1 inhibitors with chemotherapeutic agents such as paclitaxel, cisplatin, doxorubicin, and PARP inhibitors exhibits effective synergistic effects in cell proliferation and tumor growth in EOC. Phase I and II clinical trials of PLK1 inhibitors in patients with EOC have shown favorable safety but inconsistent clinical efficacy. This review analyzes the existing preclinical and clinical data on PLK1 and evaluates the antitumor effects of PLK1 in EOC, offering perspectives on the potential application of PLK1 in the treatment of EOC.

## 1 Introduction

Ovarian cancer is the most aggressive gynecological cancer, representing the sixth leading cause of cancer-related mortality among women in the USA (Siegel et al., 2025). Risk factors associated with ovarian cancer include advanced age, family history of ovarian or breast cancer, inherited genetic mutations such as BRCA1/2, and reproductive factors such as infertility, endometriosis, and polycystic ovary syndrome (Webb and Jordan, 2024). Ovarian cancer is a very heterogeneous disease that encompasses various histologic subtypes. Epithelial ovarian cancer (EOC) is the most prevalent form, making up 85%–90% of all ovarian cancer cases (Richardson et al., 2023). Despite the better prognosis for ovarian cancer diagnosed at early-stage, its insidious onset, vague symptoms, and lack of accurate early detection tests provide substantial barriers to prompt early diagnosis, resulting in more than 70% of cases being diagnosed at an advanced stage (Webb and Jordan, 2024; Smolarz et al., 2025). Primary debulking surgery followed by platinum-based chemotherapy is the standard treatment for patients with EOC. Current maintenance therapy for EOC patients is antiangiogenic drugs versus poly (ADP-ribose) polymerase (PARP) inhibitors (PARPi), especially for patients with BRCA1/2 mutations or homologous recombination deficiency (Smolarz et al., 2025; Kuroki and Guntupalli, 2020). Although the initial response rate of first-line treatment is about 75%–80%, more than 75% of patients acquire drug resistance and experience tumor recurrence (Richardson et al., 2023; Yang et al., 2022). Thus, the development of novel treatment approaches is highly warranted to enhance the survival outcome for EOC patients.

Polo-like kinase 1 (PLK1), a serine/threonine protein, exerts a vital function in various cellular events, including cell division, DNA damage response, and genome stability in eukaryotic cells (Iliaki et al., 2021; Chapagai et al., 2025). Aberrant PLK1 activity promotes cancer cell proliferation and invasion through overriding mitotic checkpoints, suppressing cell apoptosis, inducing cellular stress, and facilitating epithelial-mesenchymal transition (EMT) (Iliaki et al., 2021; Chiappa et al., 2022). PLK1 can also affect tumor cell growth by directly regulating tumor suppressor genes and oncogenes (including TP53, PTEN, KRAS, and c-MYC) through its kinase activity or by directly binding to specific domains of genes (Iliaki et al., 2021; Yim and Erikson, 2014). The Cancer Genome Atlas (TCGA) data show that PLK1 is overexpressed in more than 20 human cancers, and high expression levels of PLK1 are closely associated with aggressive tumor phenotypes, worse treatment response, and poor prognosis in colorectal cancer, bladder cancer, lung adenocarcinoma, and breast cancer (Liu et al., 2017; Guerrero-Zotano et al., 2023; Zhang et al., 2022). Inhibition of PLK1 activity by si-RNA or small molecule inhibitors demonstrates potent antitumor effects and synergizes with conventional chemotherapeutic agents in various pre-clinical models of cancer (Chapagai et al., 2025; Chiappa et al., 2022; Su et al., 2022; Zhao et al., 2025). However, multiple clinical trials of the first two generations of PLK1 inhibitors (such as BI2536, volasertib and rigosertib) have demonstrated limited clinical activity in solid tumors when used as monotherapy or in combination with other chemotherapeutic agents (Iliaki et al., 2021; Chapagai et al., 2025). The newly developed third-generation PLK1 inhibitor onvansertib is currently undergoing clinical assessment in multiple clinical trials. Preliminary results show that onvansertib combined with FOLFIRI/bevacizumab has a manageable safety profile and good efficacy in the second-line treatment of patients with KRAS-mutated metastatic colorectal cancer (Ahn et al., 2024; Ahn et al., 2025).

PLK1 plays a critical role in ovarian function by mediating oocyte meiosis; however, its expression in ovarian epithelial cells is typically low due to their limited proliferative activity (Takai et al., 2001; Weichert et al., 2004). Analysis of TCGA data revealed that PLK1 is overexpressed by more than 10-fold in EOC patients compared with normal ovarian tissues (Liu et al., 2017; Rödel et al., 2020). Targeting PLK1 significantly inhibits cell proliferation, triggers cell cycle arrest, induces DNA damage and apoptosis in EOC cells, and suppresses tumor growth while prolonging survival in animal models of EOC (Zhang et al., 2015; Affatato et al., 2020; Valsasina et al., 2012; Wang et al., 2021; Noack et al., 2018; Gasimli et al., 2022). In addition, PLK1 inhibitors such as volasertib and onvansertib exhibit potent synergistic effects with paclitaxel and PARPi via inducing strong mitotic arrest and cell apoptosis in preclinical models of EOC (Zhang et al., 2015; Affatato et al., 2020; Gasimli et al., 2022; Affatato et al., 2022). Phase I/II clinical trials were conducted to investigate PLK1 inhibitors such as volasertib and rigosertib as monotherapy in EOC patients, which demonstrated acceptable tolerability profiles and partial disease response in 15%–35% of EOC patients (Jimeno et al., 2008; Pujade-Lauraine et al., 2016). These results indicate the possibility of PLK1 inhibitors as a treatment strategy in EOC. This review is specifically focused on the role of PLK1 in EOC and the therapeutic potential of PLK1 inhibitors in preclinical and clinical studies in EOC.

## 2 The physiological function of PLK1 in normal cells

**FIGURE 1:**
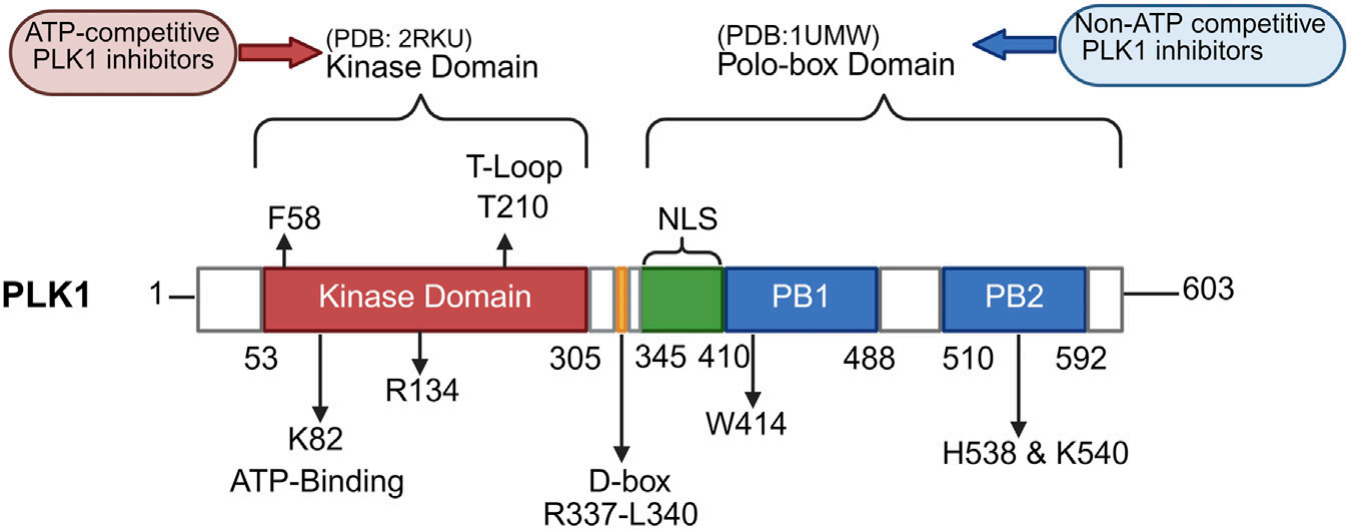
Structure of PLK1 protein and binding sites of PLK1 inhibitors. The PLK1 protein (UniProt P53350) consists of 603 amino acids, including an N-terminal kinase catalytic domain (red) and two C-terminal polo-box domains (PB1 and PB2; blue). The kinase domain (amino acids 53–305, PDB: 2RKU) includes critical phosphorylation sites, such as the P-loop/ATP pocket F58, the ATP-binding site K82, the active-site stabilizer R134, and the T-loop T210. The polo-box domains are divided into PB1 (amino acids 410–488) and PB2 (amino acids 510–592, PDB: 1UMW), with important phosphorylation sites, such as H538 and K540. ATP-competitive PLK1 inhibitors target the kinase domain, whereas non-ATP competitive PLK1 inhibitors target the polo-box domains. The data was obtained from https://www.uniprot.org/.

PLK1 was originally discovered in *Drosophila* and is an evolutionarily conserved enzyme that belongs to the PLK family (Iliaki et al., 2021; Chapagai et al., 2025). This family is essential for the cell division process and includes five members in mammals: PLK1, PLK2, PLK3, PLK4, and PLK5, each of which has the same major domains: an N-terminal kinase domain, a linker region, and a C-terminal Polo-box domain (PBD) (Goroshchuk et al., 2019). PLK1-4 contains serine/threonine residues at the N-terminus for kinase activation, whereas PLK5 contains a shorter, inactive kinase domain (Goroshchuk et al., 2019). PLK1 is the most well-studied member of the PLK family and consists of 603 amino acids with an N-terminal kinase catalytic domain (with 252 amino acids), two C-terminal PBDs (with 60–70 amino acids) (Figure 1) (Chapagai et al., 2025; De Cárcer et al., 2011). The N-terminal kinase domain is a Ser/Thr kinase domain with a T-loop whose phosphorylation status directly modulates the kinase activity of PLK1 (De Cárcer et al., 2011). Specific phosphorylation of Ser137 and Thr210 in the N-terminal kinase domain by the kinase Aurora A and its cofactor Bora is key to activating PLK1 (Iliaki et al., 2021; Kalous and Aleshkina, 2023). In addition, the phosphatidylinositol 3-kinase (PI3K) pathway promotes phosphorylation of Ser99, which is essential for mitotic entry into anaphase (Kasahara et al., 2013). The PBD domain is essential for the specific subcellular localization of PLK1 by binding to the phosphorylation sites of targeted substrates, and the genetic changes in this domain disrupt localization and impair its function (Chapagai et al., 2025; Elia et al., 2003a). In addition to subcellular localization, the PBD domain also relieves the auto-inhibitory effect on the N-terminal catalytic domain, stimulating the kinase activity in PLK1 (Elia et al., 2003b). As a serine/threonine protein kinase, PLK1 employs both self-priming and non-self-priming phosphorylation mechanisms to modify its substrates, depending on whether they have been previously phosphorylated by PLK1 itself or by other kinases (Iliaki et al., 2021). One example of self-priming of PLK1 is that PLK1 self-regulates the PLK1-PBIP1 interaction by phosphorylating PBIP1 at T78, creating a self-tethering site that specifically interacts with the PBD of PLK1 (Kang et al., 2006). In contrast, substrates like BUBR1 are first phosphorylated by CDK1, which prepares them for subsequent binding to the PBD, and then further phosphorylation by PLK1, which is essential for stabilizing kinetochore-microtubule interactions during mitosis (Elowe et al., 2007). PLK1 exerts kinase activity through self-priming and non-self-priming phosphorylation and participates in a variety of physiological and pathological processes in humans.

PLK1 is primarily enriched in mitotic centrosomes, kinetochores, and cytokinetic midbody but it can also be found in both the nucleus and cytoplasm during the S phase, G2 phase, and prophase of mitosis. The dynamics of PLK1 ensure its homeostasis and function in cell cycle progression and DNA damage response through the interaction of protein phosphatase and ubiquitination processes (Liu D. et al., 2012; Lindon and Pines, 2004). Upregulation of PLK1 begins after S phase and continues to increase during G2 phase, eventually reaching peak expression during mitosis (Schmucker and Sumara, 2014). After cell division is complete, PLK1 protein levels drop dramatically due to ubiquitin-dependent proteolysis initiated by the anaphase-promoting complex/cyclin (APC/C) CDH1 ubiquitin ligase and remain low in G1 and S phases (Lindon and Pines, 2004). This precise regulation of the expression of PLK1 reflects its primary function as a key cell cycle regulator, regulating centrosome maturation, mitotic entry, spindle formation, and cytokinesis (Gutteridge et al., 2016). During centrosome maturation, PLK1 and Aurora A kinase are cooperatively recruited to CEP192, a well-conserved scaffold protein, where PLK1 forms docking sites for γ-tubulin ring complexes, a crucial feature of centrosome maturation (Meng et al., 2015). After Aurora A binds to CEP192, PLK1 phosphonates Thr44 on CEP192 via self-priming (Meng et al., 2015). The interactions of CEP192-PLK1 are required for γ-tubulin recruitment to centrosomes and spindle formation (Iliaki et al., 2021). PLK1 also phosphorylates other centromeric proteins, such as Gravin and Cenexin, which promotes PLK1 localization to centromeres and recruitment of γ-tubulin (Soung et al., 2009). In mitotic entries, the activation of PLK1 induced by Aurora A kinase and Bora leads to the degradation of the CDK1 inhibitors WEE-1 and MYT-1 (Watanabe et al., 2005; Nakajima et al., 2003). In addition, PLK1 phosphorylates CDC25C and Cyclin B1 and promotes the nuclear localization of CDC25C at prophase during the G2/M transition, thereby activating the Cyclin B1/CDK1 complex and initiating the mitotic kinase signaling cascade. (Iliaki et al., 2021). Equal segregation of mitotic chromosomes is essential for genome integrity and is controlled by the spindle assembly checkpoint (SAC) (McAinsh and Kops, 2023). PLK1 interacts with Monopolar spindle 1 and BUB1 and promotes the formation of the Mitotic checkpoint complex, a key complex in the SAC signaling cascade that prevents the premature activation of APC/C and mitosis exit (Goroshchuk et al., 2019; Gutteridge et al., 2016). Aberrant activation of PLK1 may prematurely activate the APC/C before chromosomes are properly aligned on the mitotic spindle, causing cells to enter anaphase prematurely and leading to genomic instability (Iliaki et al., 2021). Furthermore, PLK1 serves as a key molecular for spindle assembly formation and cytokinesis via its kinase activity and loss of PLK1 leads to mitotic arrest and spindle defects, ultimately contributing to chromosome instability and prompting apoptosis (Gutteridge et al., 2016).

In addition to cell cycle regulation, PLK1 is involved in other cellular processes such as DNA replication, DNA damage response, and genome stability. DNA replication begins with the orderly assembly of the pre-replication complex (pre-RC) at the replication origin (Prasanth et al., 2004). PLK1 is able to phosphorylate several components of pre-RC, including ORC2, MCM2-7, and HBO1, which helps maintain pre-RC and promote DNA replication under stress (Song et al., 2011; Tsvetkov and Stern, 2005; Wu and Liu, 2008). PLK1 responds to DNA damage signals occurring during interphase and G2 phases by inhibiting its own phosphorylation and activation, thereby preventing cells carrying damaged genetic information from entering mitosis (Iliaki et al., 2021). When DNA damage occurs in interphase, the ATM/ATR-dependent checkpoint pathway, a crucial sensor of DNA damage, inhibits PLK1 activation by interfering with the function of Aurora A and the Bora/PLK1 complex (Qin et al., 2013). TP53 has a delicate balance with PLK1 through their negative interaction (Chen et al., 2006; Lin et al., 2011). Severe DNA damage triggers TP53 to transcription to inhibit PLK1 expression and promote cell apoptosis, while successful DNA damage repair ensures that the TP53-PLK1 axis activates PLK1 in a timely manner to promote cell cycle progression (Chen et al., 2006; Lin et al., 2011). Aberrant upregulation of PLK1 can facilitate TP53 degradation by phosphorylating the TP53 inhibitor MDM2, the TP53-interacting and -stabilizing protein NUMB, and the TP53 negative regulator GTSE1, thereby impairing TP53 function and allowing cells to continue through the cell cycle, which can lead to genomic instability and potentially cellular transformation (Dias et al., 2009; Shao et al., 2018; Liu et al., 2010).

Given the critical physiological role of PLK1 in cell cycle regulation pathways and DNA damage repair, it is not surprising that abnormal PLK1 activity is associated with tumorigenesis. Microinjection of PLK1 mRNA into NIH3T3 fibroblasts can induce tumor formation in nude mice, while transfection of PLK1 in human prostate epithelial cells can result in malignant transformation *in vitro* and promotes tumor formation in mouse models, indicating the crucial role of PLK1 in tumorigenesis (Smith et al., 1997; Wu et al., 2016). Overexpression of PLK1 can restore G2/M arrest caused by DNA damage by activating CDK1 in mammalian cells, leading to premature cell division and increased genomic instability (van Vugt et al., 2004; Degenhardt and Lampkin, 2010). PLK1 also directly exerts anti-apoptotic activities by interfering with caspase-8 auto-activation in Hela cells, decreasing cell sensitivity towards the extrinsic apoptosis pathways during mitosis (Matthess et al., 2014). Increasing the activity of PLK1 promotes tumor EMT process and enhances cancer cell invasiveness by interacting with the MEK1/2-ERK1/2-ZEB1/2, FoxM1, and cRAF signaling pathways (Iliaki et al., 2021; Fu and Wen, 2017).

## 3 Physiological role of PLK1 in the normal ovary

The ovaries are integral components of the female reproductive system and have endocrine and gametogenic functions. They are enclosed by a dense fibrous capsule of germinal epithelium, which protects the ovaries and participates in the repair process post-ovulation. Under normal conditions, ovarian epithelial cells have limited proliferative activity, hence PLK1 expression in ovarian epithelial cells is typically low (Takai et al., 2001; Weichert et al., 2004). Stimulation of primary normal ovarian epithelial cells with the estrogen analog bisphenol A (BPA) results in increased expression of PLK1, indicating that the function of PLK1 in ovarian epithelial cells may be regulated by estrogen (Zahra et al., 2022). Beneath the epithelium, the ovary tissue is histologically divided into the outer cortex and an inner medulla, and the cortex is essential for oocyte generation and endocrine function. During the menstrual cycle, progesterone secreted by ovarian granulosa cells can induce oocytes to enter meiosis by activating two distinct signal transduction pathways: the PLK1/Cdc25C pathway and the Mos/MAP kinase pathway (Gross et al., 2000). During oocyte meiosis, PLK1 is involved in regulating the nuclear envelope breakdown (NEBD), assembly of the major microtubule organizing centers (MTOCs), spindle formation, and proper chromosome segregation (Kalous and Aleshkina, 2023; Du et al., 2015; Wang et al., 2017). The PLK1 inhibitor BI2536 represses the accumulation of phosphorylated PLK1 (Ser137) at the MTOCs and disrupts meiotic spindle formation, leading to the arrest of oocytes at metaphase I with chromosome misalignment (Du et al., 2015).

## 4 Effect of PLK1 on the prognosis of ovarian cancer

**FIGURE 2:**
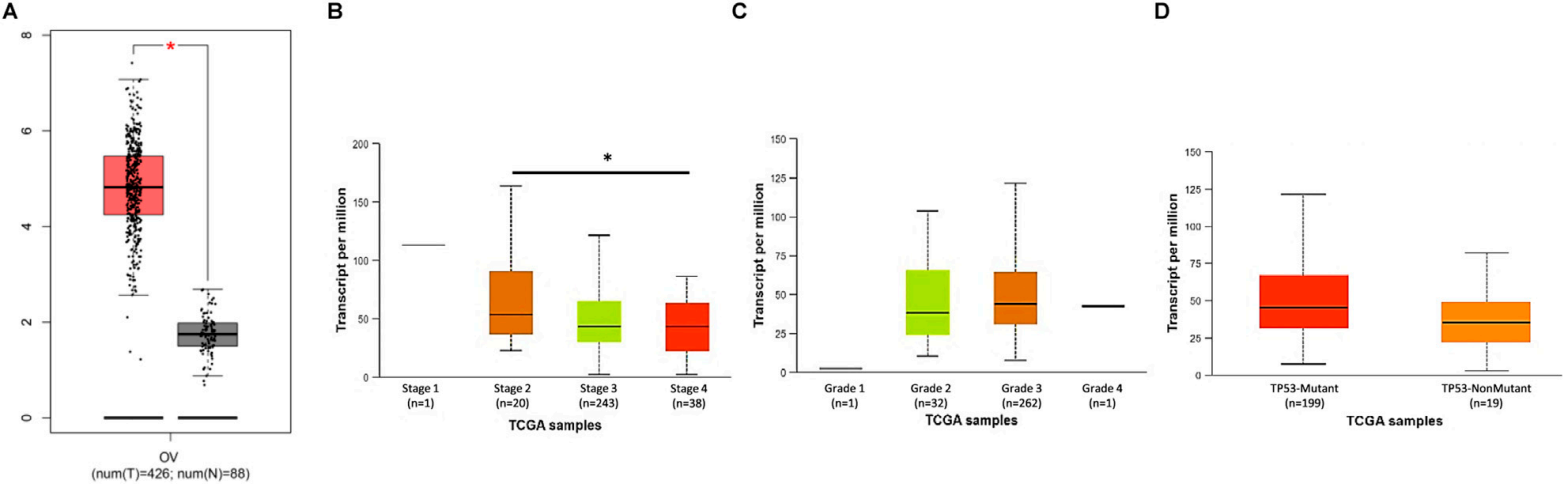
PLK1 mRNA expression in ovarian cancer. **(A)** Tumor–normal comparison generated with GEPIA (TCGA-OV tumors vs. GTEx normal ovary; log2 (TPM+1) normalization). **(B)** Stage-stratified PLK1 expression from UALCAN (TCGA-OV; log2 (TPM+1)). **(C)** Grade-stratified expression from UALCAN (TCGA-OV; log2 (TPM+1)). **(D)** TP53 status-stratified expression from UALCAN (TCGA-OV; log2 (TPM+1)). *p < 0.05.

The results from immunohistochemistry showed that the expression of PLK1 protein in EOC tissues is significantly higher than that in the normal ovarian epithelium (Takai et al., 2001; Weichert et al., 2004; Zhang et al., 2015). Using GEPIA (TCGA-OV tumors vs. GTEx normal ovary, log2 (TPM+1) normalization), we demonstrated that mRNA level of PLK1 in EOC tissues was much higher than that in normal ovarian tissues (tumor n = 426, normal n = 88, p < 0.05) (Figure 2A). Further analysis using the UALCAN portal (based on TCGA-OV RNA-seq data; log2 (TPM+1) normalization) indicated that PLK1 expression did not significantly differ across clinical stages (FIGO I–IV), histological grades (G1–G3) or TP53 status of ovarian cancer (p > 0.05 for both comparisons, except for stage 2 vs. stage 4) (Figures 2B–D). In the early 2000s, it was first found that the percentage of PLK1 expression in 17 EOC patients was positively correlated with histological differentiation and increased FIGO stage, with more de-differentiated EOC cells exhibiting stronger PLK1 staining (Takai et al., 2001). Besides, EOC cells that invaded the stroma had stronger PLK1 staining than non-invasive EOC cells (Takai et al., 2001). However, a subsequent study involving 77 EOC patients suggested that the expression of PLK1 was not associated with FIGO stage or histological differentiation but was closely related to overall prognosis (RR = 2.4, 95% CI 1.087–5.356, P = 0.03) (Weichert et al., 2004). The mortality rate of patients with PLK1 overexpression (immune response score (IRS) 7–12) was 2.4 times higher than that of patients with low PLK1 expression (IRS 0–6) (Weichert et al., 2004). Recent studies involving more than 400 patients with early-stage EOC (stage I/II) using univariable analyses have shown that patients with overexpression of PLK1 (weighted score (WS) > 6) had significantly shorter progression-free disease (PFS) and overall survival (OS) than those with the low PLK1 expression (WS < 6), and PLK1 expression (P = 0.045) remained significant independent factors in multivariate analyses (Rödel et al., 2020; Raab et al., 2019). Analysis of the TCGA database also found that the PFS [hazard ratio (HR) = 5.07 (1.8–14.27), P = 0.00063] and OS [HR = 3.6 (0.97–13.32), P = 0.04] of stage I/II EOC patients with high expression of PLK1 mRNA were significantly shorter than those of patients with low PLK1 expression, however, this conclusion was not supported in patients with the advanced stage (stage III/IV) (Rödel et al., 2020). These findings suggest that the prognostic relevance of PLK1 may be restricted to early-stage EOC, while in advanced disease its expression appears less predictive, possibly reflecting biological and treatment-related heterogeneity. However, the number of early-stage cases remains limited, and further studies with larger cohorts are needed to validate these associations. Besides, PLK1 has been proved to be involved in homologous recombination, and PLK1 expression has been positively correlated with homologous recombination deficiency (HRD) scores in various cancers, including EOC (Pae et al., 2024). Analysis of 18 ovarian cancer samples further revealed that higher PLK1 expression was associated with increased sensitivity to PARPis (Pae et al., 2024). Overall, current studies support that the overexpression of PLK1 is associated with the prognosis of early-stage EOC patients, but the relationship between PLK1 expression and prognosis in advanced-stage patients is still inconclusive although PLK1 still increased in these stages (Table 1).

**TABLE 1:** Relationship between PLK1 expression and clinicopathological features and prognosis of EOC.

Years	No. of patients	Methods	PLK1 level	Prognosis	FIGO stage	Histology	Refs
2001	27 (EOC: 17, N: 10)	IHC	Higher percentage in EOC tissues compared with normal ovary tissues	-	Stage I (10): 14.7% ± 5.8% Stage II (2): 23.0% ± 9.6% Stage III (4): 32.5% ± 9.4%* Stage IV (1):12.70%	Grade 1 (9): 13.5% ± 5.2% Grade 2 (5): 21.9% ± 8.6%^#^ Grade 3 (3): 35.0% ± 5.2%^#,a^	Takai et al. (2001)
2004	103 (EOC: 77, N: 26)	IHC, IRS	PLK1 Positive (IRS 7–12) rate: EOC: 26% N: 11.5% (p < 0.05)	PLK1 positive group have 2.4-fold higher motility rate than negative group (RR = 2.4, 95% CI 1.087–5.356, P = 0.03)	Stage I (2): 11.8% Stage II (3): 33.3% Stage III (13): 28.3% Stage IV (2): 40.0%	Grade 1 (2): 13.3% Grade 2 (8): 24.2% Grade 3 (10): 34.5%	Weichert et al. (2004)
2006	52 (EOC: 39, N: 13)	IHC, IRS	EOC: 3.87 ± 0.62 N: 0.70 ± 0.22 (p < 0.01)	-	-	-	Gao et al. (2006)
2015	91 (EOC: 52, N: 39)	IHC	EOC: 38.5% N: 2.6% (p < 0.05)	-	Stage I/II (16): 11.8% Stage III/IV (36): 50% (P = 0.014)	Grade 1 (10): 0% Grade 2–3 (42): 16.2% (P = 0.008)	Zhang et al. (2015)
2019	Stage I/II EOC: 263	IHC, WS	-	High PLK1 (WS > 6) (116): OS 62.3 months, 95%CI: 52.8–76.8 Low PLK1 (WS < 6) (147): 75.9 months, 95% CI: 68.1–83.7) (P = 0.028)	-	-	Raab et al. (2019)
2020	Stage I/II EOC: 218	IHC, WS	-	High PLK1 group (WS > 6) (95) induced impaired OS [HR 1.74 (1.09–2.7), P = 0.018] and PFS [HR 1.48 (1.02–2.16), P = 0.036] compared to low PLK1 group (WS < 6) (123)	-	-	Rödel et al. (2020)

*: p < 0.05 vs. Stage I; ^#^: p < 0.05 vs. Grade 1; ^a^: p < 0.05 vs. Grade 2. IRS: Immunoreactivity-scoring, N, normal ovary tissues, WS, weighted score.

## 5 PLK1 is a potential treatment target for epithelial ovarian cancer

The successful use of CDK4/CDK6 inhibitors in the treatment of breast cancer has attracted attention to novel therapeutic approaches targeting PLK1 to cause cell cycle arrest (Liu et al., 2017). Inhibiting PLK1 has shown multifaceted antitumor effects in cancer cells, while its transient expression during the cell cycle progression can minimize its impact on normal cells with low proliferative activity (Su et al., 2022). Multiple clinical trials have demonstrated the safety profile of PLK1 inhibitors, such as volasertib, onvansertib, and rigosertib, in patients with advanced cancer, and have shown potential antitumor activity in combination with certain conventional chemotherapeutic agents (Iliaki et al., 2021). Moreover, significant progress has been made recently in developing new PLK1 inhibitors with higher specificity and favorable pharmacokinetics (Zhang et al., 2022; Stafford et al., 2023). Considering the huge obstacles faced in the treatment of EOC, recent investigations have increasingly focused on evaluating the antitumor effects of PLK1 in EOC, particularly its potential synergistic effects with conventional therapeutic agents.

### 5.1 Targeting PLK1 exerts antitumor effects

Research using shRNA screening has pinpointed PLK1 as an essential gene for the survival of EOC cells, with these cells exhibiting heightened vulnerability to PLK1 inhibition compared to non-cancerous cells (Valsasina et al., 2012; Kim et al., 2016). Treatment of human dermal fibroblasts and EOC A2780 cells with the same dose of onvansertib for 8 h leads to mitotic block and DNA damage in both cell types (Valsasina et al., 2012). However, after the removal of onvansertib, dermal fibroblasts fully recovered cell viability, but A2780 cells continued to undergo mitotic block and eventually cell death, indicating the potential selectivity of PLK1 inhibitors (Valsasina et al., 2012). Despite the great heterogeneity of EOC, PLK1 inhibitors from different generations or PLK1 siRNA significantly reduce cell proliferation and invasiveness, induce G2/M arrest, causing DNA damage and activating apoptosis and autophagy in EOC cells (Noack et al., 2018; Chan et al., 2018; Gorski et al., 2020; Belur et al., 2018). However, certain molecular features or cisplatin-resistance status in EOC cells can affect the sensitivity of EOC cells to PLK1 inhibition. CCNE1 is essential for G1/S phase transition, and its amplification is a common genetic alternation and a primary oncogenic driver in EOC (Gorski et al., 2020). Overexpression of CCNE1 in EOC cells significantly enhances cell growth inhibition by PLK1 inhibitors through the PLK1/FBW7/CCNE1 axis (Noack et al., 2018; Gorski et al., 2020; Xi et al., 2025). For example, the IC50 value of volasertib was reduced more than threefold in OVCAR3 and COV318 cells with CCNE1 amplification compared to Ovsaho cells without CCNE1 amplification (Noack et al., 2018). Similarly, EOC cells harboring HSF1–c-MYC co-amplification exhibited much greater sensitivity to PLK1 inhibition compared to cells with wild-type HSF1 and c-MYC (Williams et al., 2025). A recent study identified three genes—JUND, CARD9, and BCL2L2—as synthetic lethal partners of onvansertib in EOC using a CRISPR/Cas9 library. Hence, the combination of navitoclax, a BCL2 family inhibitor targeting BCL2L2, showed synergistic cytotoxicity in all EOC cell lines tested (Petrella et al., 2025). Furthermore, cisplatin-resistant EOC cells have mitotic exit dysfunction caused by APC/C dysregulation, which makes these cells heavily dependent on another mitotic exit regulator, PLK1, for mitotic exit and cell survival (Belur et al., 2018). Therefore, cisplatin-resistant EOC cells are often accompanied by higher PLK1 expression and increased sensitivity to PLK1 inhibitors. Treatment of cisplatin-resistant EOC cells with volasertib or si-RNA of PLK1 showed greater inhibition of cell proliferation in cisplatin-resistant cells compared with cisplatin-sensitive EOC cells (Belur et al., 2018), supporting a link between platinum-resistance and PLK1 signaling.

Treatment of the SKOV3 xenograft mouse model of EOC with volasertib (10 mg/kg, intravenously, every 3 days, for 6 times) or the A2780 xenograft mouse model of EOC with onvansertib (60 mg/kg, orally) for 10 consecutive days significantly inhibited tumor growth in both mouse models and increased the expression of PLK1 in SKOV3 tumor tissues compared with control mice (Valsasina et al., 2012; Wang et al., 2021). Time course analysis of tumor tissues showed that a single oral treatment with 3 escalating doses of onvansertib (60, 90, 120 mg/kg) blocked cell mitosis for 6–12 h in a dose-dependent manner in A2780 xenograft mice, indicating that onvansertib has unique PLK1 selectivity *in vivo* (Valsasina et al., 2012). Recently, several novel PLK1 inhibitor delivery systems have been developed to improve efficiency and reduce toxicity. Volasertib, via its effects on folate-targeted and α3 integrin-targeted polymersomes, effectively increases drug deposition, enhances inhibition of tumor growth, and reduces toxicity in SKOV3 xenograft mice compared with volasertib alone (Wang et al., 2021; Wang et al., 2022a; Wang et al., 2022b). Overall, the current data supports the inhibitory role of targeting PLK1 in EOC *in vivo* and *in vitro*, but whether some specific molecular backgrounds (such as CCNE1 overexpression) or biological states (such as cisplatin resistance) affect the efficacy of PLK1 in tumor growth deserves to be investigated in animal models of EOC.

### 5.2 Targeting PLK1 improves sensitivity to chemotherapeutic agents

Paclitaxel serves as the first-line chemotherapy for ovarian cancer treatment, which stabilizes and prevents microtubule depolymerization, leading to cell cycle arrest at the G2/M phase and cell death (Yang and Horwitz, 2017). However, most EOC patients eventually develop chemoresistance, resulting in disease recurrence or death (Kampan et al., 2015; Tossetta, 2022). The ability of PLK1 to regulate microtubule dynamics suggests that it may have a synergistic inhibitory effect on tumor cell growth when used in combination with paclitaxel. PLK1 inhibitors and paclitaxel showed synergistic inhibitory effects on tumor growth in preclinical models of breast cancer and osteosarcoma, and PLK1 expression was upregulated when OVCAR3 cells were treated with increasing concentrations of paclitaxel (Noack et al., 2018; Liu X. S. et al., 2012; Li et al., 2010; Chou et al., 2016). It is speculated that this combination therapy may also have similar synergistic effects in patients with EOC. The combination of volasertib and paclitaxel showed obvious synergistic antitumor effects through various mechanisms such as causing cell apoptosis, inducing mitotic arrest, and preventing mitotic exit of EOC cells (Noack et al., 2018; Raab et al., 2019). CCNE1 amplification in EOC cells appears to be a trigger for activating this synergistic antitumor activity, as this combination therapy showed significant synergistic effects in CCNE1-amplified EOC cells compared with CCNE1-nonamplified cells (Noack et al., 2018). The combination of onvansertib or volasertib with paclitaxel also resulted in a significant decrease in cell viability, increased apoptosis, and DNA damage in mucinous EOC cell lines and demonstrated more effective tumor growth inhibition and prolonged survival in xenograft animal models of EOC compared with single-agent treatment (Affatato et al., 2020). Another study investigating three different PDX models of EOC with TP53 mutations and platinum resistance demonstrated that a three-week combination of paclitaxel (15 mg/kg, IV, weekly) and onvansertib (50 mg/kg, oral, 4 days per week) prolonged median survival by more than 1.3-fold and 2.4-fold, respectively, in two of three models compared with paclitaxel and onvansertib-alone (Affatato et al., 2022). In terms of safety, the combination of paclitaxel and onvansertib was well-tolerated, with a weight loss no greater than 15% compared with the control group (Affatato et al., 2022).

Although PARPis have been used for maintenance therapy of EOC patients and effectively prolong OS, some patients fail to benefit from PARPi due to acquired drug resistance (Jiang et al., 2019). Aberrant KRAS expression has been implicated in PARPi resistance in ovarian cancer cells and xenograft mouse models of EOC (Sun et al., 2017; Yang B. et al., 2021). In BRCA2-deficient KURAMOCHI cells with KRAS amplification and PARPi resistance, sequential therapy with volasertib followed by olaparib proved to be more effective than the reverse sequence (olaparib followed by volasertib), whereas in BRCA2-deficient OVSAHO cells without KRAS mutations, different sequential therapy demonstrated similar effect on cell proliferation, suggesting that volasertib before PARPi may help reverse PARPi resistance in BRCA-deficient KRAS-amplified EOC cells (Gasimli et al., 2022). Another study also demonstrated the combination of onvansertib and olaparib has addictive or synergic antitumor effects in different EOC cell lines and statistically increased survival in olaparib-resistant-BRCA1 mutated PDX models of EOC (Chiappa et al., 2024). Onvansertib was also active, although to a lesser extent, in the BRCA1 wild-type PDX models (Chiappa et al., 2024). All these data support the clinical evaluation of onvansertib with PARPis in EOC. In addition, the expression level of PLK1 affects the sensitivity of EOC cells to doxorubicin. In doxorubicin-sensitive OVCAR8 cells and multidrug-resistant NCI/ADR-RES cells, knocking down PLK1 by si-RNA significantly increased their sensitivity to doxorubicin through a TP53-mediated mechanism, with a 10-fold decrease in the IC50 in OVCAR8 cells and a 20-fold decrease in the IC50 in NCI/ADR-RES cells (Benoit et al., 2010). Furthermore, onvansertib or volasertib demonstrated strong synergistic inhibition of cell proliferation with the EGFR inhibitor eribulin and the PI3K inhibitor PIK75 in three EOC cell lines (MCAS, JHOM1, and EFO27 cells) using an isobologram model based on the MTS assay (Affatato et al., 2020).

**FIGURE 3:**
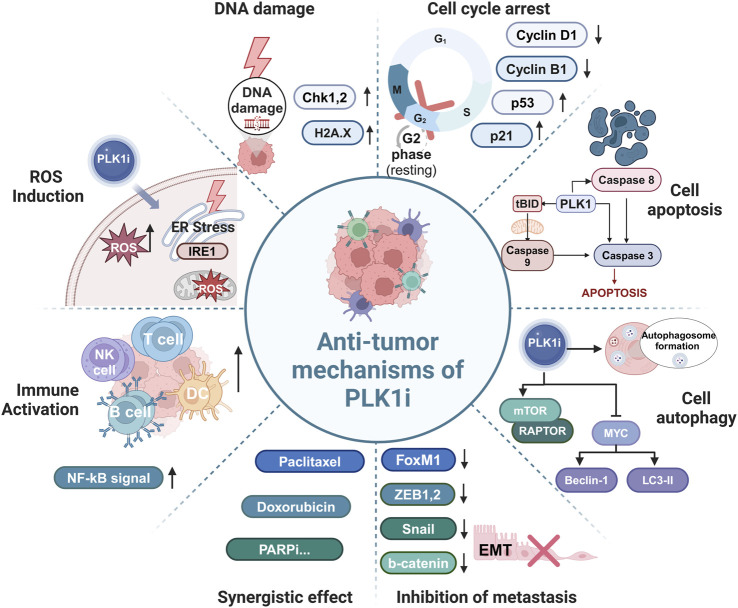
Antitumor mechanisms of targeting PLK1. Inhibition of PLK1 exerts antitumor effects through multiple mechanisms, including causing G2/M phase arrest, inducing DNA damage and apoptosis, increasing reactive oxygen species (ROS) levels, reducing migration and invasion, enhancing immune cell infiltration and activation, and synergistically inhibiting cell proliferation and tumor growth when combined with chemotherapeutic drugs.

Several studies have explored the potential synergistic effects of PLK1 inhibitors in combination with platinum-based therapies in EOC. Knockdown of PLK1 via siRNA significantly enhanced the antitumor efficacy of cisplatin by modulating autophagy and apoptosis in EOC cells, while exogenous PLK1 transfection partially reversed cisplatin-induced apoptosis compared to scramble controls (Chan et al., 2018). The combination of carboplatin with a PLK1 inhibitor and a PARPi significantly enhanced the inhibition of cell viability in three patient-derived EOC 3D spheroids, BRCA2-deficient OVSAHO cells, and KURAMOCHI cells compared with either single-agent or double-agent groups (Gasimli et al., 2022). However, recent studies have found that cisplatin attenuates the cytotoxic effects of volasertib in cisplatin-resistant PEO4 cells compared with volasertib alone (Kim et al., 2016). In contrast, cisplatin-sensitive PEO1 cells, which are homologous to PEO4 cells, did not show the same antagonistic effects when treated with a combination of volasertib and cisplatin (Kim et al., 2016). The differential response between PEO4 and PEO1 cells may be attributed to the restoration of BRCA2 function and enhanced drug efflux mechanisms in PEO4 cells compared to PEO1 cells, but further investigation is necessary to confirm this (Cooke et al., 2010; Chu et al., 1990) (Figure 3; Table 2).

**TABLE 2:** Preclinical studies on PLK1-targeted therapy for ovarian cancer.

Cell lines	Genetic background	Drug	Experimental methods	IC50	Synergy	*In vivo* model	Outcomes	Refs
A2780	TP53-WT	Onvansertib	CellTiter-Glo assay Colony formation assay Cytofluorimetric analysis	0.042 mM	-	Nude mice (n = 4) Orally (60, 90, or 120 mg/kg) for 10 consecutive days	Inhibit cell proliferation; Inhibit tumor growth and blocked cell mitosis for 6–12 h in a dose-dependent manner *in vivo*	Valsasina et al. (2012)
SKOV3	TP53 loss, Her-2 amplified	Volasertib and A3-Ps-Volasertib	MTT assay Flow cytometry for cell cycle and apoptosis	Volasertib:172 nM; A3-Ps-Volasertib: 49 nM	-	Nude mice (n = 5) IV (10 Vol mg/kg), every 3 days, for 6 times	Inhibit cell proliferation, cause G2/M arrest, and apoptosis Inhibit tumor growth A3-Ps-Volasertib (80%) and Volasertib (50%)	Wang et al. (2021)
OVCAR3	TP53 loss, CCNE1 amplified and BRCA- WT	Volasertib	Cell Titer-Blue Cell Viability Assay; Caspase-3/7-activity Cell cycle assay	40 nM	Paclitaxel	-	Volasertib + paclitaxel shows synthetic lethality in CCNE1-amplified HGSOC cells: mitotic arrest → BCL-2 family inactivation → caspase activation, with FBW7 stabilization regulating MCL-1/Cyclin E	Noack et al. (2018)
KK and OVTOKO	KK: ARID1A-WT OVTOKO: ARID1A-mut	si-PLK1	Flow cytometry for cell cycle and apoptosis MTT, BrdU incorporation and clonogenic assays; Sphere forming culture	-	Cisplatin	-	PLK1 inhibition heightened chemosensitivity to cisplatin partially via the regulation of autophagic flux	Chan et al. (2018)
OV81.2 OV231 SKOV3	OV81.2: NR OV231: NR SKOV3: TP53 loss, Her-2 amplified	si-PLK1 and Volasertib	MTT and clonogenic assay; Annexin V-PI Staining; Cell cycle assay	Effect dose Volasertib 50 nM	-	Nude mice (n = 5) si-PLK1 cells injection	Cisplatin-resistant EOC exhibit APC/C dysfunction → mitotic-exit vulnerability with PLK1 dependence. PLK1 inhibition reduces cell survival *in vitro*/*in vivo*, and APC/C^CDC20 blockade further sensitizes	Belur et al. (2018)
OVCAR3 OVISE OVTOKO JHOC5	OVCAR3 OVISE: CCNE1-high expression OVTOKO JHOC5:CCNE1-low expression	Volasertib	CCK8 assay Cell cycle analysis Time-lapse microscopy for apoptosis detection IHC	OVCAR3 89 nM OVISE 22 nM OVTOKO: 311 nM JHOC5: 619 nM	-	Nude mice (n = 8) Orally (60 mg/kg daily) for 22 days	CCNE1 overexpression predicts heightened sensitivity to PLK1 inhibitor; Volasertib suppress tumor growth *in vivo* of CCNE1-overexpressing tumors	Xi et al. (2025)
-	#266R ((BRCA1/2 NA) #76 (BRCA1/2 WT) #124R (BRCA1/2 WT)	Onvansertib	Caspase-3 activity assay Western blot assay	-	Paclitaxel	3 patients derived PDX models Orally (50 mg/kg, 4 days a week) for 3 weeks	Onvansertib + Paclitaxel was well tolerated and more effective than either agent alone. *Ex vivo* tumors showed upregulation ofγ-H2AX and pSer10-H3 in combination group	Affatato et al. (2022)
OVSAHO KURAMO-CHI, and patient-derived 3D spheroids	BRCA2-deficient	Volasertib	Colony formation assay; 3D-Cultures; Caspase-3/7-activity; Cell cycle analysis; Immunofluorescence Assays	Effect dose: 20 nM	Olaparib	-	In BRCA2-deficient HGSOC, PLK1 inhibition restores PARPi sensitivity; sequential PLK1i→PARPi markedly reduces survival and increases apoptosis, and enhances carboplatin responses in resistant cell lines and patient-derived 3D spheroids	Gasimli et al. (2022)
OVCAR3 SKOV3	OVCAR3: TP53 loss, CCNE1 amplified and BRCA- WT SKOV3: TP53 loss, Her-2 amplified	Volasertib	Colony formation assay; Cell Titer-Blue assay; 3D-Cultures; Caspase-3/7-activity; Cell cycle analysis	OVCAR3: 15 nM SKOV3: 28 nM	Paclitaxel	-	Paclitaxel + Volasertib + APC/C inhibitor proTAME induces synergistic apoptosis by stabilizing Cyclin B1 to sustain mitotic arrest→ inactivating anti-apoptotic BCL-2 proteins and activating caspases	Raab et al. (2019)
OVCAR8 CAOV3	OVCAR8: HSF1–MYC co-amplified CAOV3: MYC–HSF1 WT	Volasertib	CellTiter-Blue and Clonogenic growth assay	OVCAR8: 19.8 nM CAOV3: 4900 nM	-	-	Volasertib exhibits >200-fold greater potency in HSF1–MYC co-amplified cells vs. wild type, supporting HSF1–MYC co-amplification as a precision biomarker for PLK1-targeted therapy	Williams et al. (2025)
MCAS, EFO27, and TOV2414	MCAS: KRAS-mut、PIK3CA-mut EFO27: TP53 mut TOV2414: KRAS-mut	Onvansertib	CRISPR/Cas9; Caspase Activity; Cell Cycle/Annexin V	Effect dose: 75 nM	Navitoclax	-	In mEOC, a CRISPR/Cas9 (3,015 genes) screen ± onvansertib identified 3 synthetic lethal partners (JUND, CARD9, BCL2L2); Onvansertib + navitoclax (targets BCL2 family) was synergistic across mEOC lines	Petrella et al. (2025)
SKOV3	TP53 loss, Her-2 amplified	si-PLK1and Volasertib FA-Ps-si-PLK1 and FA-Ps-Volasertib	Flow cytometry; MTT assay; Cell apoptosis and Western blot measurements; *In vivo* imaging	Volasertib: 193 nM	-	Nude mice (n = 5) Tail vein injection, FA-Ps-Vol (20 Vol mg/kg) FA-Ps-siPLK1 (2–4 mg/kg), every 3 days, for 6 times	In xenografts, FA-Ps-Vol: TIR 97% with reduced toxicity; FA-Ps-siPLK1: TIR 87%, Volasertib 65%	Wang et al. (2022a)
SKOV3	TP53 loss, Her-2 amplified	HER2-targeted volasertib (Tra-PVol)	MTT assay *In vivo* imaging	Volasertib: 239 nM Tra-PVol: 50 nM	-	Orthotopic SKOV-3-Luc tumor-bearing mice (n = 6) IV or IP (20 Vol mg/kg) on day 0,3,6,9	Tra-PVol shows enhanced uptake and potency in HER2-high SKOV-3 cells, superior tumor deposition (IP > IV) *in vivo*, suppresses intraperitoneal metastasis, prolongs survival, and maintains low toxicity versus Vol	Wang et al. (2022b)
OVCAR3 ES2	OVCAR3: TP53 loss, CCNE1 amplified and BRCA- WT ES-2: TP53 loss, BRCA- WT, PALB2 mutated	Onvansertib	MTS assay; Flow cytometry analyses for cell cycle and apoptosis; Caspase 3/7 activity assay; Immunofluorescence assay	Effect dose: OVCAR3 30 nM ES2 50 nM	Olaparib	PDX model (n = 8) (All models are TP53 mutated with both acquired or intrinsic olaparib resistance) Orally (50 mg/kg 5 days a week for 4 weeks	Onvansertib + Olaparib showed additive/synergistic effect *invitro* and *in vivo* (stronger in BRCA1-mutant). Mechanism: onvansertib impairs HR and NHEJ, functionally inducing HR deficiency and enhancing PARPi activity	Chiappa et al. (2024)
NCI/ADR-RES OVCAR8	NCI/ADR-RES: MDR cells OVCAR8: drug-sensitive cells	si-PLK1	Cell viability and caspase 3/7 assay	-	pH-responsive ternary complexes	-	pH-responsive ternary complexes + siPLK1 sensitizing MDR cells to doxorubicin via p53-dependent apoptosis	Benoit et al. (2010)

A3-Ps, Integrin targeted polymersomes; FA-Ps, Folate-targeted polymersomes; HR, homologous recombination; IHC, immunohistochemistry; IV, intravenously; IP, intraperitoneal; MDR, multiple drug resistance; mEOC, mucinous epithelial ovarian cancer; NR, not consistently reported; TIR, tumor inhibition rate; WT, Wild-type.

## 6 Role of PLK1 inhibitors in clinical trials of EOC

Currently, there are two common types of PLK1 inhibitors: ATP-competitors (targeting KD) and non-ATP competitors (targeting PBD), both of which have entered clinical trials in hematologic malignancies and solid tumors because of their promising antitumor effects of PLK1 in pre-clinical models of cancer (Figure 1; Table 3). The initial phase I clinical trials investigating PLK1 inhibitors as a single drug, such as ATP-competitors BI 2536 and non-ATP competitors ON 01910 (rigosertib), began at the early 2000s, but the development of these two drugs in solid tumors was eventually discontinued due to low response rates (Schöffski et al., 2010; Mross et al., 2012; Sebastian et al., 2010; O'Neil et al., 2015). The second-generation ATP-competitor inhibitor volasertib, which belongs to the dihydropteridinone class like BI2536, has improved pharmacokinetic attributes and has been granted Breakthrough Therapy status designation by the U.S. Food and Drug Administration (FDA) for the treatment of acute myeloid leukemia (AML), given that this agent has been shown to prolong OS and PFS in combination with low-dose cytarabine (LDAC) in AML patients aged over 65 years in a phase II clinical trial (Döhner et al., 2014). Volasertib has limited phase II clinical data in solid tumors and has not significantly improved OS or PFS, either as a monotherapy or in combination with other treatments (Ellis et al., 2015; Stadler et al., 2014). The third-generation ATP-competitive PLK1 inhibitor onvansertib is the most specific and selective PLK1 inhibitor and can be administered orally, rather than intravenously like the other PLK1 inhibitors (Valsasina et al., 2012). Several ongoing phase I/II clinical trials are investigating the antitumor efficiency of onvansertib as an adjuvant therapy for hematologic and solid tumors (Zhang et al., 2022; Ahn et al., 2024; Ahn et al., 2025). A phase II clinical trial of AML demonstrated that the combination of onvansertib with decitabine had promising antitumor results, particularly in patients with SRSF2 mutations (Croucher et al., 2023). Another Phase Ib clinical trial of onvansertib combined with FOLFIRI and bevacizumab showed a manageable safety profile and a promising response rate compared with the historical response rate to FOLFIRI/bevacizumab in patients with KRAS-mutant metastatic colorectal cancer (mCRC) (Ahn et al., 2024). A subsequent multicenter, open-label, single-arm phase II trial showed that onvansertib plus FOLFIRI/bevacizumab was effective in second-line treatment of KRAS-mutant mCRC, especially with notably higher objective response rate (ORR, 76.9%) and median PFS (14.9 months) in patients without prior exposure to bevacizumab (Ahn et al., 2025). These promising results have prompted the ongoing evaluation of onvansertib in the first-line setting, where all enrolled patients are bevacizumab-naïve (Ahn et al., 2025).

**TABLE 3:** Clinical trials of PLK1 inhibitors in solid tumors.

PLK1 inhibitors	Classifications	Routes	Clinical trials status	Cancers (No. of patients/ No. With EOC)	Strategies	Outcomes	Main grade ≥3 adverse events	NCT No.	Refs
BI 2536	ATP- competitive	IV	Phase I	Advanced solid tumors (40)	Monotherapy	Tolerable safety profile. MDT: 200 mg. SD was found in 23% of patients	Neutropenia (45%) and Leukopenia (35%)	Not reported	Mross et al. (2008)
				NSCLC (41)	Adjuvant therapy (Pemetrexed)	RP2D: 200 mg plus pemetrexed. SD was found in 54% of patients	Neutropenia (54%) and Anemia (12%)	NCT02211833	Ellis et al. (2013)
			Phase II	SCLC (23)	Monotherapy	Terminated for a lack of efficacy	Anemia (43%) and Neutropenia (39%)	NCT00412880	Awad et al. (2017)
				Pancreatic cancer (86)	Monotherapy	Low ORR (2.3%) and poor survival were observed	Neutropenia (36.1%) and Leukopenia (28%)	NCT00710710	Mross et al. (2012)
				NSCLC (95)	Monotherapy	PR was found in 4.2% of patients	Neutropenia (35.4%)	NCT00376623	Sebastian et al. (2010)
				Advanced solid tumors (71)	Monotherapy	No objective responses were observed in five different tumor types	Febrile neutropaenia (2.8%) and Fatigue (2.8%)	NCT00526149	Schöffski et al. (2010)
Volasertib (BI6727)	ATP- competitive	IV	Phase I	Advanced solid tumors (65)	Monotherapy	Tolerable safety profile. RP2D: 300 mg. 40% of patients achieved SD.	Neutropenia (14%) and Thrombocytopenia (14%)	Not reported	Schöffski et al. (2012)
				Advanced solid tumors (15)	Monotherapy	Tolerable safety profile. MTD in Japanese patients: 300 mg	Neutropenia (53%), Leukopenia (33%), and Thrombocytopenia (33%)	NCT01348347	Nokihara et al. (2016)
				Advanced solid tumors (57)	Adjuvant therapy (Afatinib)	MTD: volasertib 300 mg plus afatinib 30 mg. SD was found in 28% of patients and PR was found in 9% of patients	Neutropenia (39.3%), Thrombocytopenia (35.7%), and Hypokalemia (14.3%)	NCT01206816	Machiels et al. (2015)
				Advanced solid tumors (30)	Adjuvant therapy (Nintedanib)	MTD: volasertib 300 mg plus nintedanib. 60% of patients achieved disease control (CR + PR + SD)	Neutropenia (50%), Thrombocytopenia (30%), Increased ALT (23%), and Increased AST (17%)	NCT01022853	De Braud et al. (2015)
				Advanced solid tumors (61)	Adjuvant therapy (Cisplatin or Carboplatin)	MTD: volasertib 300 mg plus cisplatin 100 mg/m^2^ or carboplatin AUC 6	Neutropenia (43.3%), Lymphopenia (30%), Fatigue (20%), and Leukopenia (20%)	NCT00969761	Awada et al. (2015)
			Phase II	Ovarian cancer (109)	Monotherapy	Volasertib achieved equivalent PFS compared to single-agent non-platinum cytotoxic chemotherapy (hazard ratio, 1.01; 95% CI, 0.66–1.53)	Neutropenia (44.4%), Leukopenia (16.7%), and Thrombocytopenia (16.7%)	NCT01121406	Pujade-Lauraine et al. (2016)
				Urothelial cancer (50)	Monotherapy	Antitumor activity of volasertib was insufficient to warrant further evaluation as a monotherapy	Neutropenia (28%), Thrombocytopenia (20%), and Anemia (16%)	NCT01023958	Stadler et al. (2014)
				NSCLC (131)	Adjuvant therapy (Pemetrexed)	Volasertib plus pemetrexed did not increase PFS compared to the pemetrexed group (hazard ratio 1.141; 95% CI 0.73–1.771)	Neutropenia (10.9%)	NCT00824408	Ellis et al. (2015)
GSK461364	ATP- competitive	IV	Phase I	Advanced solid tumors (40/1)	Monotherapy	Tolerable safety profile.15% of patients achieved SD.	Venous thrombotic emboli (20%)	NCT00536835	Olmos et al. (2011)
Onvansertib (NMS-1286937)	ATP- competitive	Oral	Phase I	Advanced solid tumors (19)	Monotherapy	Tolerable safety profile. MTD and RP2D were 24 mg/m^2^/day. 31% of patients achieved SD. 60% of patients with SD were KRAS-mutant tumors	Thrombocytopenia (15.8%) and Neutropenia (15.8%)	NCT01014429	Weiss et al. (2018)
			Phase Ib	KRAS-mutant mCRC (18)	Adjuvant therapy (FOLFIRI/bevacizumab)	RP2D: 15 mg/m^2^ plus FOLFIRI/bevacizumab. PR was confirmed in 44% of patients, with a median duration of response of 9.5 months. Further exploration of this combination therapy is ongoing	Neutropenia (55%) and Leukopenia (23%)	NCT03829410	Ahn et al. (2024)
			Phase II	KRAS-mutant mCRC (53)	Adjuvant therapy (FOLFIRI/bevacizumab)	ORR (CR + PR): 26.4% (95% CI, 15.3–40.3); Disease control rate (CR + PR + SD): 92.5%; PFS: 8.4 months (95% CI 6.0–14.8) Bevacizumab-naïve group had a significantly higher ORR (76.9%, 95% CI 46.2–95.0) and longer PFS (14.9 months, 95% CI 13.5 to not reached) compared to those with prior exposure (6.6 months, 95% CI 5.6–9.8) (hazard ratio 0.16, P < 0.001)	Neutropenia (41.5%) and Hypertension (9.4%)	NCT06106308	Ahn et al. (2025)
Rigosertib (ON 01910.Na)	Non-ATP competitive	Oral	Phase I	Advanced solid tumors (48)	Monotherapy	Tolerable safety profile. RP2D: 560 mg twice daily. CR and PR were observed in 2 patients with head and neck SCC.	Dysuria (4%)	NCT01168011	Bowles et al. (2014)
Rigosertib (ON 01910.Na)	Non-ATP competitive	IV	Phase I	Advanced solid tumors (25)	Monotherapy	Tolerable safety profile. RP2D: 2400 mg. 36% of patients achieved SD.	Anemia (8%)	Not reported	Advani et al. (2014)
				Advanced solid tumors (20/3)	Monotherapy	Tolerable safety profile. RP2D: 3120 mg. PR was observed in one refractory ovarian cancer patient	Skeletal (5%)	Not reported	Jimeno et al. (2008)
			Phase II/III	Pancreatic cancer (40)	Adjuvant therapy (GEM)	RP2D: rigosertib 1800 mg/m^2^ and GEM 1000 mg/m^2^. SD was found in 58% of patients and PR was found in 6% of patients	Neutropenia (27.5%) and Thrombocytopenia (15%)	NCT01125891	Ma et al. (2012)
				Pancreatic cancer (153)	Adjuvant therapy (GEM)	No improvement in survival in the rigosertib + GEM group compared with GEM alone	Hyponatremia (17%)	NCT01360853	O'Neil et al. (2015)

CI, Confidence interval CR, complete response; GEM, gemcitabine; IV, intravenous; mCRC, metastatic colorectal cancer; MTD, maximum tolerated dose; NSCLC, Non-small cell lung cancer; ORR, objective response rate; PFS, Progression-free survival; PK, pharmacokinetics; PR, partial response; RP2D, Recommended phase II; dose; SCC, squamous cell carcinomas; SCLC, small cell lung cancer; SD, stable disease; TNBC, triple negative breast cancer.

PLK1 inhibitors exhibit relatively consistent hematologic toxicities, particularly neutropenia, thrombocytopenia, and anemia, which are the most common dose-limiting toxicities; neutropenia can occur in approximately 25%–40% of patients, highlighting the importance of close monitoring of hematology parameters in clinical application (Ahn et al., 2024; Jimeno et al., 2008; Pujade-Lauraine et al., 2016; de Braud et al., 2015). In a phase Ib clinical trial investigating onvansertib in patients with solid tumors, grade 3/4 adverse events (AEs) accounted for 15% of all reported AEs, with neutropenia (28%) and leukopenia (17%) identified as the most common hematologic toxicities associated with onvansertib treatment (Ahn et al., 2024). Diarrhea (50%), nausea (50%), and fatigue (44%) were the most common non-hematologic adverse reactions associated with onvansertib (Ahn et al., 2024). More importantly, when PLK1 inhibitors are used in combination with other drugs such as cisplatin, paclitaxel, and doxorubicin in the clinical trials, they do not significantly affect the pharmacokinetics of these chemotherapeutic drugs or increase the toxicity of these chemotherapeutic drugs (Su et al., 2022; de Braud et al., 2015). Given that hematologic toxicities are the most common dose-limiting events, routine complete blood count monitoring is recommended at baseline and at least weekly during treatment and prophylactic granulocyte colony-stimulating factor (G-CSF) is advised for patients at high risk of neutropenia. Nevertheless, further well-designed clinical studies are needed to better define optimal monitoring schedules and mitigation strategies for PLK1 inhibitors in different patient populations.

Clinical studies of PLK1 inhibitors in EOC are still in their initial stages. In 2008, a phase I clinical trial involving 20 cancer patients (3 with ovarian cancer) evaluated the potential antitumor activity of Rigosertib, the only non-ATP competitor PLK1 inhibitor that is also a known RAS inhibitor and PI3K inhibitor (Iliaki et al., 2021; Jimeno et al., 2008). Of the three EOC patients treated with rigosertib, one patient showed an obvious decrease in CA125 levels and tumor volume, achieved a partial response, and remained progression-free for 24 months, while the other two patients either experienced disease progression or discontinued treatment due to grade 5 adverse events (Jimeno et al., 2008). In 2011, a phase I Study of GSK461364 included a patient with advanced EOC patient, whose disease was stable for 19 weeks. This study also found that patients with tumors harboring high mitotic activity may benefit more from treatment with PLK1 inhibitors (Olmos et al., 2011). In a randomized phase II trial of 109 patients with platinum-resistant or refractory EOC in 2016, volasertib achieved equivalent antitumor activity to single-agent non-platinum cytotoxic chemotherapy in terms of PFS and disease control rate (Pujade-Lauraine et al., 2016). However, among the patients treated with volasertib, 6 patients (11%) had a prolonged PFS of more than 1 year but none in the chemotherapy group, indicating volasertib may be beneficial for some heavily pretreated patients with resistant/refractory ovarian cancer (Pujade-Lauraine et al., 2016). Although PLK1 inhibitors combined with paclitaxel or PARPi have synergistic effects in inhibiting tumor cell growth in preclinical models of EOC, there are currently no reports on the combination of PLK1 inhibitors with other chemotherapeutic drugs in clinical trials of EOC. Future trials could investigate the combinations such as volasertib or onvansertib with paclitaxel or olaparib, particularly in patients with high PLK1 expression, CCNE1 amplification, or HR-proficient/BRCA–wild-type tumors. Companion diagnostics assessing PLK1 and CCNE1 expression and HRD/BRCA status may help guide patient selection. PFS and objective response rate should be listed as key endpoints and monitoring for hematologic toxicity by complete blood count should be conducted during and after each treatment cycles. Overall, biomarker-driven phase Ib/II studies are warranted to validate these strategies in the future.

## 7 Discussion

Although several PLK1 inhibitors have shown promising antitumor effects in preclinical studies, these agents were ultimately not used in clinical practice due to low therapeutic response rates in clinical trials. Indeed, the application of PLK1 inhibitors has certain limitations, including the high sequence homology among PLK family members, off-target kinase effects, the complex role of PLK1 in cell cycle regulation, and the cell cycle-dependent expression of PLK1, all of which collectively lead to challenges in inhibitor selectivity, toxicity control, development of drug-resistance, and effectively targeting of slowly proliferating tumors (Chapagai et al., 2025; Stafford et al., 2023). Structure-guided medicinal chemistry offers an opportunity to develop more selective ATP-competitive inhibitors to overcome these challenges by targeting unique PLK1 residues within the ATP-binding pocket, such as F58 and R134 (Park et al., 2017). The development of dual ATP-competitive inhibitors that recognize dual targets, such as PLK1-BRD4, PLK1-NEK2, and PLK1-WEE1 inhibitors, has become a recent trend in the development of PLK1 inhibitors targeting the kinase domain (Zhang et al., 2022). Meanwhile, a recent study found that ATP-competitive inhibitors may induce an open but catalytically inactive conformation of PLK1, thereby enhancing the non-catalytic functions mediated by the PBD, which may partly account for their limited clinical efficacy (Chapagai et al., 2025). This further supports the rationale for targeting the PBD domain as a complementary strategy (Chapagai et al., 2025). With the identification of several key binding sites of the PBD domain of PLK1, such as the broad pyrrolidine-binding pocket, the deep tyrosine-rich channel, and the phosphate-binding pocket, as well as the PLK1-specific residues (L478, L491, R516, and F535), non-ATP competitive PLK1 inhibitors developed against the unique PBD domain of PLK have shown great potential for reducing nonspecific cross-reactivity and inhibiting tumor growth in preclinical models (Zhang et al., 2022; Stafford et al., 2023). Furthermore, novel protein degradation strategies, including proteolysis-targeting chimeras (PROTACs), hydrophobic tagging, and lysosome-based degradation, and new drug delivery systems, such as nanoparticle-based delivery systems and exosome-based delivery, further offer innovative approaches for the development of PLK1 inhibitors (Zhang et al., 2022; Stafford et al., 2023).

Recent studies have found that changes in certain biomarkers can alter the sensitivity of cancer cells to PLK1 inhibitors (Chiappa et al., 2022; Croucher et al., 2023; Wang and Simon, 2013) (Table 4). Lung cancer cells with wild-type TP53 exist resistance to PLK1 inhibitor GSK461364A via activating a postmitotic tetraploid checkpoint and arrest in a pseudo-G1 state following PLK1 inhibition, while RNA silencing of TP53 increased the antitumor activity of GSK461364A (Degenhardt et al., 2010). The status of TP53 has been considered as a determining factor for the sensitivity of PLK1 inhibitors in combination with radiotherapy for the treatment of non-small cell lung cancer and colorectal cancer (Van den Bossche et al., 2019; Sur et al., 2009). BRCA1-deficient breast cancer cells demonstrated higher PLK1 activity, and PLK1 inhibitors trigger stronger synthetic lethality in BRCA1-deficient breast cancer cells compared with BRCA1-proficient one (García et al., 2020). Furthermore, PLK1 showed a synthetic lethal interaction with the KRAS function in colorectal cancer cells based on genome-wide RNAi screen (Luo et al., 2009). c-MYC amplification has also been shown to increase the sensitivity of PLK1 inhibitors in glioma and medulloblastoma (Higuchi et al., 2018; Valinciute et al., 2023). Transfection of c-MYC into glioma cells with low c-MYC expression not only upregulated PLK1 expression but sensitized glioblastoma cells to volasertib (Higuchi et al., 2018). Consistently, co-amplification of HSF1 and c-MYC significantly sensitized cells to PLK1 inhibition in EOC (Williams et al., 2025). All these data suggest that patient selection based on these genetic profiles may become a strategy for clinical trials of PLK1 inhibitors in the future. The molecular characteristics of EOC patients are complex, including somatic mutations of TP53 in more than 50% of EOC tumors and alterations in BRCA1/2, KRAS, and PTEN, which may make some EOC patients more vulnerable to PLK1 inhibitor treatment (Kossaï et al., 2018). However, this hypothesis needs to be verified in future clinical trials in patients with EOC.

**TABLE 4:** Biomarkers influencing the sensitivity of tumor cells to PLK1 inhibitors.

Biomarkers	Status	Sensitivity	Cancers	Evidence level	Refs
TP53	Mutation, Deletion	Increase	Lung cancer	*In vitro*	Degenhardt et al. (2010), Van den Bossche et al. (2019)
	Deletion	Increase	Colorectal cancer	*In vitro* and *in vivo* (n = 5)	Sur et al. (2009)
	Mutation	Increase	Adrenocortical carcinoma	*In vitro*	Warmington et al. (2024)
KRAS	Mutation	Increase	Colon cancer Lung cancer Pancreatic cancer	*In vitro* and *in vivo* (n = 5) *In vitro* and *in vivo* (n = 5) *In vitro* and *in vivo* (n = 8)	Luo et al. (2009), Yang Z. et al. (2021), Wang et al. (2016)
c-MYC	Amplification	Increase	Glioma Medulloblastoma	*In vitro* *In vitro*	Higuchi et al. (2018), Valinciute et al. (2023)
BRCA1/BRCA2	Deficiency	Increase	Breast cancer	*In vitro* and *in vivo* (n = 5)	García et al. (2020), Carbajosa et al. (2019)
CCNE1	Amplification	Increase	Ovarian cancer	*In vitro*	Noack et al. (2018)
PTEN	Deletion	Increase	Prostate cancer	*In vitro* and *in vivo* (n = 5)	Luo and Liu (2012), Liu et al. (2011)

## 8 Limitations

Although current preclinical and clinical data support the potential therapeutic value of targeting PLK1 in epithelial ovarian cancer, several limitations should be acknowledged. First, potential publication bias may exist in the preclinical literature, as studies with positive findings are more likely to be published than those reporting negative or inconclusive results. This bias may lead to an overestimation of the efficacy of PLK1 inhibition. Second, most of the clinical trials conducted to date are early phase with small sample sizes and late-stage disease, and they enroll heterogeneous populations with respect to tumor histology, prior therapies, and molecular profiles, which complicates the interpretation of clinical outcomes and limits the applicability of the findings to specific ovarian cancer subtypes. Third, hematologic toxicities such as neutropenia and thrombocytopenia remain the most common dose-limiting adverse events of PLK1 inhibitors and may restrict their use in combination with other myelosuppressive agents, including platinum and paclitaxel.

## 9 Conclusion

The role of PLK1 is essential for oocyte meiosis under the regulation of sex hormones. Overexpression of PLK1 in EOC tissues has been shown to be closely related to the prognosis of patients with early-stage EOC, but its impact on the prognosis of patients with advanced stage remains to be further elucidated. Targeting PLK1 via siRNA or PLK1 inhibitors demonstrates potential antitumor activity and enhanced synergy with chemotherapeutic drugs such as paclitaxel, doxorubicin, and PARP inhibitors in preclinical models of EOC. While initial phase I/II trials of PLK1 inhibitors in EOC patients have demonstrated a favorable safety profile, their effectiveness as monotherapy remains inconsistent, and there is a lack of data on combining PLK1 inhibitors with other chemotherapeutic agents. As recent studies have identified certain molecular biomarkers that may influence the antitumor activity of PLK1 in solid tumors, molecular genetic signatures unique to EOC may help optimize the selection of certain EOC patients and potentially improve treatment outcomes.
